# Differential Expression of Genes Involved in Saikosaponin Biosynthesis Between *Bupleurum chinense* DC. and *Bupleurum scorzonerifolium* Willd

**DOI:** 10.3389/fgene.2020.583245

**Published:** 2020-10-16

**Authors:** Ma Yu, Hua Chen, Shi-Hang Liu, Yu-Chan Li, Chun Sui, Da-Bin Hou, Jian-He Wei

**Affiliations:** ^1^Institute of Medicinal Plant Development (IMPLAD), Chinese Academy of Medical Sciences and Peking Union Medical College, Beijing, China; ^2^School of Life Sciences and Engineering, Southwest University of Science and Technology, Mianyang, China; ^3^Triticeae Research Institute, Sichuan Agricultural University, Chengdu, China

**Keywords:** *Bupleurum*, saikosaponin biosynthesis, transcriptome analysis, gene expression, P450

## Abstract

Radix Bupleuri (roots of *Bupleurum* spp.) is an important medicinal herb. Triterpenoid saponins of saikosaponins generally constitute the main class of secondary metabolites of plants in the *Bupleurum* genus. However, the molecular regulatory mechanism underlying their biosynthesis remains elusive. In this study, we observed significantly different saikosaponin biosynthesis between *Bupleurum chinense* and *Bupleurum scorzonerifolium* at the seedling stage. The sequential and expression characterization of 232 genes in the triterpenoid saponin biosynthetic pathway, which includes the mevalonate (MVA) pathway and methylerythritol phosphate (MEP) pathway, between *B. chinense* and *B. scorzonerifolium* was also investigated. Sixty of these genes may be involved in saikosaponin biosynthesis. Manipulation of these genes, especially those of the β-AS, P450, and UGT families, may improve saikosaponin production.

## Introduction

Radix Bupleuri (roots of *Bupleurum* spp.) is one of the most important medicinal herbs in Eurasia and North Africa used as a treatment for fever, chronic hepatitis, nephrotic syndrome, inflammatory diseases, menstrual disorders, and digestive ulcers (Pistelli et al., 1996; Guo et al., 2000; Ikegami et al., 2006; Mabberley, 2008). In addition, this herb has been used for more than 2,000 years in China (Tan et al., 2008). In the Chinese Pharmacopoeia, the official botanical origin of Bupleuri Radix is the roots of *Bupleurum chinense* DC. or *Bupleurum scorzonerifolium* Willd. (Chinese Pharmacopoeia Commission, 2015). Several groups of secondary metabolites have been isolated from *Bupleurum* species, including triterpenoid saponins (saikosaponins), steroidal saponins, lignans, essential oils, and polysaccharides (Ashour and Wink, 2011). Among these, saikosaponins generally represent the main class of secondary metabolites and constitute to up to 7% of the total dry weight of roots of plants in the *Bupleurum* genus (Ashour and Wink, 2011). Owing to their wide range of pharmacological activities, including their immunomodulatory activity, anti-inflammatory activity, antioxidant and hepatoprotective activity, cytotoxicity, antitumor activity, and antiviral activity, these triterpenoid saponins, especially saikosaponins a and d, are the most important pharmacological constituent in *Bupleurum* root extracts (Zhao and Xiao, 2007; Lin et al., 2013; Liang et al., 2014).

To date, more than 130 glycosylated oleanane-type and ursane-type saponins have been isolated from the genus *Bupleurum* L. (Ashour and Wink, 2011; Wang et al., 2017b; Chelghoum et al., 2018). A previous study on the species *B. chinense*, *B. scorzonerifolium*, and *Bupleurum falcatum* L. demonstrated that saikosaponins are mainly distributed in the tissues of the cork and cortex of roots (Liang et al., 2014). Similar results were reported based on histochemical studies (Tan et al., 2008). In addition, the synthesis and accumulation of saikosaponins is strongly influenced by intrinsic factors, including growth stage, developmental phase, and root structure, and by environmental conditions, such as drought, fertility, and light deficiency (Tan et al., 2008; Zhu et al., 2009a,b; Gong et al., 2017). The combination of cultural practices together with manipulation of the expression of genes involved in triterpenoid saponin biosynthesis may be a more effective way to improve the total yield of saikosaponins.

In the biosynthetic pathway of triterpenoid saponins in higher plants, the mevalonate (MVA) pathway in the cytosol and the methylerythritol phosphate (MEP) pathway in the plastids are essential biosynthetic processes for formation of the triterpenoid backbone of the five-carbon intermediates isopentenyl diphosphate (IPP) and dimethylallyl diphosphate (DMAPP). In transcriptome studies, genes involved in the biosynthetic pathway of saikosaponins were identified in *Bupleurum kaoi*, *B. chinense*, and *B. scorzonerifolium* (Chen et al., 2007; Sui et al., 2011; Sui et al., 2015). Moreover, genes involved in the biosynthesis of saikosaponins such as squalene epoxidase, β-amylase, cytochrome P450, and uridine diphosphate glycosyl transferases have been cloned in *B. kaoi*, *B. falcatum*, and *B. chinense*, and their expression profiles have been identified (Lin et al., 2006; Kim et al., 2011; Gao et al., 2016). Overexpression of the *BcbZIP134* gene in *B. chinense* and *BfSS1* in *B. falcatum* has been reported (Kim et al., 2011; Xu et al., 2019). Additionally, cytochrome P450 monooxygenase (P450) of *CYP716Y1* from *B. falcatum* was combined with oxidosqualene cyclase, P450, and glycosyltransferase genes to construct a synthetic biological platform for the production of bioactive triterpene sapo(ge)nins in yeast (Moses et al., 2014). However, the molecular regulatory mechanism underlying triterpenoid saponin biosynthesis remains elusive.

In this study, we investigated the sequential and expression characterization of 232 genes in the MVA pathway and MEP pathway in *B. chinense* and *B. scorzonerifolium* to identify putative genes involved in the biosynthesis of saikosaponins in *Bupleurum* L.

## Materials and Methods

### Plant Materials

The two experimental materials, the commercial varieties Chuanbeichai No. 1 (CBC1) and Chuanhongchai No. 1 (CHC1), which are varieties of *B. chinense* and *B. scorzonerifolium*, respectively, were used. All of the plants were bred via systemic selection and purifying selection from farmholding populations by Dr. Jianhe Wei from the Institute of Medicinal Plant Development (IMPLAD), Chinese Academy of Medical Sciences and Peking Union Medical College, and Dr. Da-bin Hou from Southwest University of Science and Technology.

For each genotype, seeds were placed on moist filter paper before germination. Germinated seedlings were then grown in modified Hoagland’s nutrient solution. Five-day-old and 15-day-old CBC1 and CHC1 plants were utilized for isoform sequencing (iso-seq) analysis, transcriptome analysis, and saikosaponin a (SS a) and d (SS d) content assays. For 5-day-old seedlings, whole fresh roots were harvested (S1). For 15-day-old seedlings, 5 mm of the root tip without the region of differentiation (S2) and with the region of differentiation (S3) were harvested separately.

### Extraction of Saikosaponins and HPLC Analysis

Samples S1, S2, and S3 (three replications each) were dried for 72 h using a freeze-drier (LGJ-18, Beijing Songyuan Huaxing Technology Development Co., Ltd., China). The SS a and SS d content was determined using a Waters HPLC (high-performance liquid chromatography) system (Waters 1525 Binary HPLC Pump, United States) and an ASB-vensil C18 column (250 mm × 4.6 mm, 5 μm). Reference standards of SS a and SS d were purchased from the National Institutes for Food and Drug Control, Beijing, China. The methods and conditions for determination have been reported previously (Xu et al., 2019).

### Iso-seq and Transcriptome Analyses

The leaf and root samples were mixed and utilized for iso-seq library construction. The iso-seq analysis followed the method published by Wang et al. (2017a). Transcriptome analyses of samples S1, S2, and S3 were performed on an Illumina HiSeq 2500 platform (Illumina, San Diego, CA, United States) as previously described (Yang et al., 2018). Both iso-seq and transcriptome analyses were performed at the Novogene Bioinformatics Institute (Novogene, Beijing, China). Three replications were included in this study.

### Candidate Gene Selection

Gene sequences of 20 families involved in the MVA pathway and MEP pathway were selected from iso-seq and transcriptome data with annotated gene names. These genes include glycosyltransferases (UGT), P450, β-amyrin synthase (β-AS), squalene synthase (SS), squalene epoxidase (SE), farnesyl diphosphate synthase (FPS), mevalonate-5-pyrophosphate decarboxylase (MVD), phosphomevalonate kinase (PMK), mevalonate kinase (MK), 3-hydroxy-3-methylglutaryl-CoA reductase (HMGCR), 3-hydroxy-3-methylglutaryl-CoA synthase (HMGS), acetyl-CoA C-acetyltransferase (AACT), 1-deoxy-D-xylulose-5-phosphate synthase (DXS), 1-deoxy-D- xylulose-5-phosphate reductoisomerase (DXR), 2-C-methyl-D-erythritol 4-phosphate cytidylyl transferase (CMS), 4-(cytidine 5′-diphospho)-2-C-methyl-D-erythritol kinase (CMK), 2-C-methyl-D-erythritol-2,4-cyclodiphosphate synthase (MCS), 4-hydroxy-3-methyl but-2-(E)-enyl diphosphate synthase (HDS), 4-hydroxy-3-methyl but-2-(E)-enyl diphosphate reductase (IDS), isopentenyl diphosphate isomerase (IDI), and geranyl diphosphate synthase (GPS). CAP3 software was used to identify overlaps between different sequences and to remove redundant fragments^1^. The conserved domains of each family were analyzed by using SMART online software^2^ and used as a query to search the NCBI non-redundant protein database via BLASTX to further validate the potential candidate genes^3^. The isoelectric points (PIs) and molecular weights (MWs) of deduced proteins were calculated using the ExPASy Compute pI/Mw tool^4^, and the subcellular localizations were predicted using Cell-PLoc 2.0^5^. Differential expression analyses were performed by the DESeq2 R package (Anders and Huber, 2010) to identify differentially expressed genes (DEGs) among S1, S2, and S3 within each species and between the two species. The differentially expressed unigenes were further filtered based on their count number (at least one stage was greater than 1) and the log2(fold change) [the log2(fold change) between two stages was greater than 2].

## Results

SS a and SS d were detected in the roots of *B. chinense* plants at the 1-day-old stage, whereas they significantly accumulated in the region of differentiation at the 15-day-old stage (Table 1). In *B. scorzonerifolium*, no peaks of SS a and SS d were identified in any of the samples during the HPLC analysis. Therefore, the genes showed significantly different expression in S3 in *B. chinense*, but insignificant or the opposite expression in *B. scorzonerifolium* would be interesting.

**TABLE 1:** Contents of saikosaponin a and saikosaponin d in the roots of *B. chinense* and *B. scorzonerifolium.*

		SS a		SS d	
		content		content	
Species	Sample	(μg/g)	SD	(μg/g)	SD
*B. chinense*	S1	0	0	0	0
	S2	0	0	0	0
	S3	479.77	154.95	270.64	154.34
*B. scorzonerifolium*	S1	0	0	0	0
	S2	0	0	0	0
	S3	0	0	0	0

**FIGURE 1:**
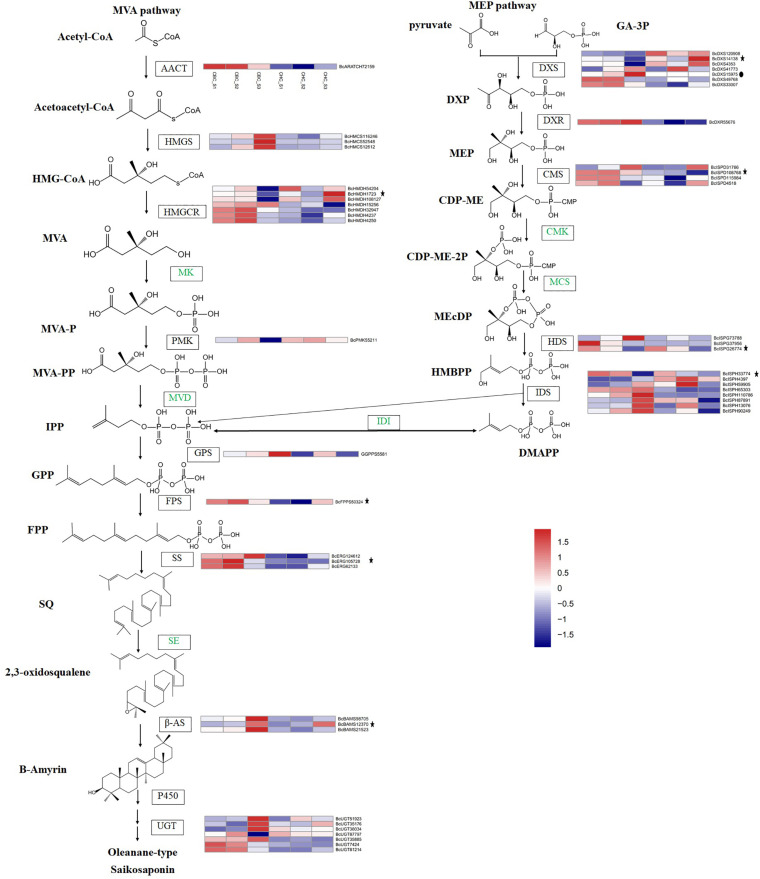
Putative triterpenoid backbone biosynthesis pathway (MVA and MEP) in *Bupleurum chinense* DC. and *Bupleurum scorzonerifolium* Willd. and gene expression profiles of the key enzymes. All genes outside of these pathways showed significantly differential expression in S3 of *B. chinense*. The genes with asterisks also showed significantly differential expression in S3 of *B. scorzonerifolium*. The genes with solid dots indicate those detected only in *B. chinense* DC. The samples include S1 (F), S2 (T), and S3 (M) in *B. chinense* (CBC) and *B. scorzonerifolium* (CHC).

**FIGURE 2:**
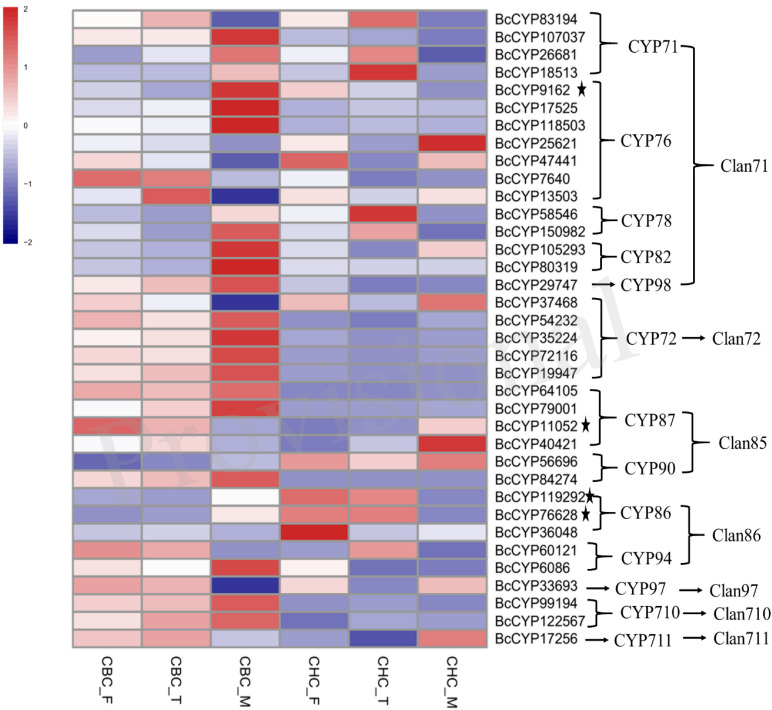
Gene expression profiles of candidate P450s involved in the biosynthesis of triterpenoid saponins. All these genes showed significantly differential expression in S3 of *Bupleurum chinense* DC. The genes with asterisks also showed significantly differential expression in S3 of *Bupleurum scorzonerifolium* Willd. The sample information is listed in Figure 1.

A total of 223 genes with complete open reading frames (ORFs) were identified from the transcriptome database of *B. chinense* and *B. scorzonerifolium* (Supplementary Table S1). One to 96 genes were identified for a single gene family whose members are involved in triterpenoid saponin biosynthesis, although the MCS family had zero genes. In the differential expression analysis of *B. chinense*, 86 genes showed significantly different expression in the S3 sample (Figures 1, 2). Among these genes, 1 to 36 genes were identified for gene families including UGT, P450, β-AS, SS, FPS, GPS, PMK, HMGCR, HMGS, AACT, IDS, HDS, CMS, DXR, and DXS. No gene with significantly different expression was detected for SE, MVD, IDI, MK, and CMK. Five genes involved the gene families β-AS, SS, CMS, HDS, and IDS; these genes exhibited significantly similar expression in *B. scorzonerifolium.* Seven genes in the FPS, HMGCR, DXS, and P450 gene families showed significantly reversed expression. The *BcDXS15975* gene in the DXS family was identified only in *B. chinense* and was not detected in any of the samples of *B. scorzonerifolium.*

## Discussion

Triterpenoids are derived from C5 isoprene units of IPP and DMAPP through a “head-to-tail” connection (Hillier and Lathe, 2019). Both of these triterpenoid backbones can be synthesized through the MVA pathway in the cytoplasm or the MEP pathway in the plastids. For the MVA pathway, three HMGS genes and one HMGCR gene (*BcHMDH15256*) showed significantly upregulated expression in S3 of the *B. chinens*e transcriptome, whereas one AACT gene, seven HMGCR genes, and one PMK gene showed significantly downregulated expression. For these genes, the expression differences among S1, S2, and S3 were insignificant in *B. scorzonerifolium*, with the exception of the *BcHMDH1723* gene (Figure 1). In addition, *BcHMDH1723* showed the opposite expression trend between *B. chinens*e and *B. scorzonerifolium*. We did not detect genes with significantly different expression in the MK family or the MVD family.

For the MEP pathway, 12 unigenes encoding enzymes (five DXS genes, three CMS genes, two HDS genes, and two IDS genes) showed significantly downregulated expression in S3 of the *B. chinens*e transcriptome, whereas the expression of two DXS genes, one DXR gene, one CMS gene, one HDS gene, and six IDS genes significantly increased. The CMS gene *BcISPD108768*, the HDS gene *BcISPG26774*, and the IDS gene *BcISPH33774* showed similar trends in the *B. scorzonerifolium* transcriptome, whereas the DXS gene *BcDXS14138* showed the opposite trend. The DXS gene *BcDXS15975* was detected only in the *B. chinens*e transcriptome.

Both the MVA and MEP pathways produce the C5 unit IPP, which can be transformed into its isomer DMAPP by IDI (Xue et al., 2019). In the present study, we did not find that the IDI gene showed significant expression differences among S1, S2, and S3. IPP and DMAPP are assembled into GPP and FPP by GPS and FPS prenyltransferases, respectively. Expression of the GPS gene *BcGGPPS5581* increased in S3 of the *B. chinens*e transcriptome, and expression of the FPS gene *BcFPPS50324* decreased. SS catalyzes the joining of two units of FPP in a “tail-to-tail” fashion of SQ. Kim et al. (2011) found that overexpression of *BfSS1* in *B. falcatum* more powerfully regulates downstream genes than does MeJA treatment in triterpene and phytosterol biosynthesis. In the present study, three candidate genes in the SS family showed significant expression differences in S3 of the *B. chinens*e transcriptome, and the expression of all these candidate genes among the three samples in the *B. scorzonerifolium* transcriptome was not significant. SQ is oxidized by SE to give rise to 2,3-oxidosqualene. We cloned the full-length SE gene in *B. chinens*e (Gao et al., 2016). However, no candidate gene was identified in the differential expression analysis.

In *Bupleurum*, the first committed step in the synthesis of triterpenoid saponins involves the cyclization of 2,3-oxidosqualene by β-AS. Therefore, β-AS is presumed to be the enzyme that catalyzes the first committed step in saikosaponin biosynthesis. A previous study indicated that β-AS in *B. kaoi* and *B. chinense* exhibits tissue-specific responses to both MeJA and PEG (Lin et al., 2013; Zhang et al., 2016). We have also cloned the promoter of β-AS in *B. chinens*e and evaluated its activity previously (Gao et al., 2015). We found three β-AS genes (*BcBAMS98705*, *BcBAMS12370*, and *BcBAMS21523*) with significantly increased expression in S3 of *B. chinens*e. The *BcBAMS12370* gene showed a similar expression trend in *B. scorzonerifolium*, whereas the expression of the other two genes was non-significant.

For the production of saikosaponins in *Bupleurum*, P450 enzymes catalyze the oxidation of β-amyrin to form 13,28-epoxy and the C11/C12 double-bond structure or two double bonds at C11/C12 and C13/C18 together with hydroxylation at C16 and C23. Previous studies have indicated that the CYP90, CYP72, CYP710, and CYP711 subfamilies of the P450 family may involve triterpenoid-metabolizing enzymes (Ohnishi et al., 2006; Hamberger and Bak, 2013; Yu et al., 2017). We found that 37 P450 genes showed significant expression in the three samples of *B. chinens*e, and four of these genes exhibited a significant opposite trend in *B. scorzonerifolium* (Figure 2). These genes are members of 13 families of 7 clans in the P450 family. Among these genes, two unigenes belong to the CYP90 family, five unigenes belong to the CYP72 family, two unigenes belong to the CYP710 family, and the unigene *BcCYP17256* belongs to the CYP711 family. Members of the CYP716 family contribute to the diversification of eudicot triterpenoid biosynthesis (Miettinen et al., 2017). Moses et al. (2014) found that the *CYP716Y1* gene from *B. falcatum* catalyzes the C-16α hydroxylation of triterpenes in yeast. However, we did not identify any CYP716 gene that showed significantly different expression in S3 of *B. chinens*e.

At the last step of saikosaponin biosynthesis, UGT catalyzes the glycosylation of hydroxylated β-amyrin at C3. Glycosylation contributes to the highly diverse nature of terpenoids in plants (Field and Osbourn, 2008). Therefore, UGT is presumed to be the key enzyme involved in the modification of saikosaponins. A transcriptome analysis of *B. chinense* revealed that, among 196 UGT genes, three were the most likely candidates involved in saikosaponin biosynthesis (Sui et al., 2011). Three *BkUGT85A* proteins that contain a highly conserved region of a motif of glycosyltransferases, which are involved in the production of secondary metabolites in plants, were identified in *B. kaoi* by Lin et al. (2013). In the present study, four UGT genes showed significantly increased expression in S3 of *B. chinens*e, whereas four genes showed the opposite expression trend. The expression differences among all genes in *B. scorzonerifolium* were non-significant.

## Conclusion

In this study, unigenes showed significantly different expression in the S3 sample in *B. chinense*, while insignificant or the opposite expression in *B. scorzonerifolium* may be involved in saikosaponin biosynthesis. Manipulation of these genes, especially those of the β-AS, P450, and UGT families, may improve saikosaponin production. The combined expression of these genes and reconstituting the synthesis of monoglycosylated saponins in yeast may provide a platform for the production of bioactive saikosaponins.

## Data Availability Statement

The datasets generated for this study can be found in NCBI PRJNA645610 for *B. chinense* (SRR12223465-SRR12223473) and PRJNA662700 for B. scorzonerifolium (SRR12647620-SRR12647628).

## Author Contributions

MY wrote the manuscript. HC performed the data analysis. S-HL seeded Chuanbeichai No.1 and Chuanhongchai No.1. Y-CL performed the HPLC analysis. CS performed the transcriptome analysis. J-HW bred out the commercial varieties of Chuanbeichai No.1 and Chuanhongchai No.1. J-HW and D-BH funded the whole project and helped MY to complete the manuscript. All authors contributed to the article and approved the submitted version.

## Conflict of Interest

The authors declare that the research was conducted in the absence of any commercial or financial relationships that could be construed as a potential conflict of interest.

## Footnotes

1 http://doua.prabi.fr/software/cap3

2 http://smart.embl-heidelberg.de/

3 http://www.ncbi.nlm.nih.gov/

4 http://web.expasy.org/compute_pi/

5 http://www.csbio.sjtu.edu.cn/bioinf/Cell-PLoc-2/
